# A comparative analysis of HIV drug resistance interpretation based on short reverse transcriptase sequences versus full sequences

**DOI:** 10.1186/1742-6405-7-38

**Published:** 2010-10-15

**Authors:** Kim Steegen, Michelle Bronze, Elke Van Craenenbroeck, Bart Winters, Koen Van der Borght, Carole L Wallis, Wendy Stevens, Tobias F Rinke de Wit, Lieven J Stuyver

**Affiliations:** 1 Department of Infectious Disease and Biomarkers, Tibotec-Virco Virology BVBA, Beerse, Belgium; 2 Department of Molecular Medicine and Hematology, University of the Witwatersrand, Johannesburg, South Africa; 3 Department of Research Informatics and Integrative Genomics, Tibotec-Virco Virology BVBA, Beerse, Belgium; 4 Department of Clinical Virology, Tibotec-Virco Virology BVBA, Beerse, Belgium; 5 Department of Molecular Medicine and Hematology, Faculty of Health Sciences, University of the Witwatersrand and National Health Laboratory Services, Johannesburg, South Africa; 6 Department of Health Intelligence, PharmAccess Foundation and Amsterdam Institute for Global Health and Development, Academic Medical Center, University of Amsterdam, Amsterdam, The Netherlands; 7 Contract Laboratory Services, Johannesburg, South Africa; 8 Amsterdam Institute for Global Health and Development, Academic Medical Center, University of Amsterdam, Amsterdam, The Netherlands; 9 Centre de Recherche Public de la Santé, Luxemburg; 10 PharmAccess Foundation, Amsterdam, The Netherlands; 11 University Medical Center Utrecht, Department of Virology; Utrecht, The Netherlands; 12 Tibotec-Virco Virology BVBA, Beerse, Belgium; 13 Wits Health Consortium, University of the Witwatersrand, Johannesburg, South Africa

## Abstract

**Background:**

As second-line antiretroviral treatment (ART) becomes more accessible in resource-limited settings (RLS), the need for more affordable monitoring tools such as point-of-care viral load assays and simplified genotypic HIV drug resistance (HIVDR) tests increases substantially. The prohibitive expenses of genotypic HIVDR assays could partly be addressed by focusing on a smaller region of the HIV reverse transcriptase gene (RT) that encompasses the majority of HIVDR mutations for people on ART in RLS. In this study, an *in silico *analysis of 125,329 RT sequences was performed to investigate the effect of submitting short RT sequences (codon 41 to 238) to the commonly used virco^®^TYPE and Stanford genotype interpretation tools.

**Results:**

Pair-wise comparisons between full-length and short RT sequences were performed. Additionally, a non-inferiority approach with a concordance limit of 95% and two-sided 95% confidence intervals was used to demonstrate concordance between HIVDR calls based on full-length and short RT sequences.

The results of this analysis showed that HIVDR interpretations based on full-length versus short RT sequences, using the Stanford algorithms, had concordance significantly above 95%. When using the virco^®^TYPE algorithm, similar concordance was demonstrated (>95%), but some differences were observed for d4T, AZT and TDF, where predictions were affected in more than 5% of the sequences. Most differences in interpretation, however, were due to shifts from fully susceptible to reduced susceptibility (d4T) or from reduced response to minimal response (AZT, TDF) or vice versa, as compared to the predicted full RT sequence. The virco^®^TYPE prediction uses many more mutations outside the RT 41-238 amino acid domain, which significantly contribute to the HIVDR prediction for these 3 antiretroviral agents.

**Conclusions:**

This study illustrates the acceptability of using a shortened RT sequences (codon 41-238) to obtain reliable genotype interpretations by virco^®^TYPE and Stanford algorithms. Implementation of this simplified protocol could significantly reduce the cost of both resistance testing and ARV treatment monitoring in RLS.

## Introduction

In most developed countries, HIV treatment monitoring guidelines recommend regular viral load (VL) testing and HIV drug resistance (HIVDR) testing in the case of virologic failure and prior to treatment initiation [1,2]. In contrast, current clinical practice in resource-limited settings (RLS) is predominantly based on clinical staging and/or CD4 measurements [3]. However, the latest WHO recommendations promote strategic introduction of VL monitoring as well as greater access to CD4 testing for treatment initiation[4]. In 2003 WHO and UNAIDS initiated a public health approach to HIV management by recommending standardized antiretroviral (ARV) treatment regimens in order to improve the access to HIV treatment in RLS [5]. This approach has been successful and the number of patients on treatment in low- and middle-income countries has since increased 10-fold to more than 4 million at the end of 2008 [6]. Despite these joint efforts, laboratory tools to monitor patients on treatment are still lacking in many parts of the world, due to the lack of infrastructure and financial resources.

Several studies have shown that CD4 measurements are inaccurate in predicting treatment failure [7-11], which has resulted in the aforementioned WHO recommendations. Therefore it is of utmost importance to develop simple and affordable alternatives to the currently available VL and HIVDR tests that could be better implemented in RLS. In the context of these challenges a public-private consortium, aiming to bring an affordable HIV monitoring algorithm to Africa (ART-A: affordable resistance testing for Africa) was established in 2008 with partners in South-Africa, Luxembourg, the Netherlands and Belgium [12]. The overall aim of the ART-A project is to develop a more affordable HIV treatment monitoring system which can be universally applied for both individual patient management and public health purposes. In order to achieve this, the project will look at the use of dried blood spots and combine this with a cost-effective qualitative VL testing and subtype-independent confirmatory HIVDR genotyping with automated base-calling software to reduce operator errors in identifying pure mutations and mixture mutations. One strategy to reduce the costs of HIVDR testing is to focus on a partial region of the HIV-1 reverse transcriptase (RT) from codon 41 to 238. This region covers all HIVDR mutations recognized by the IAS [13]. This approach can be justified because 98% of the patients on treatment in RLS receive a first-line drug regimen based on RT-inhibitors only [6]. Moreover, the mutations, commonly present in patients failing a first-line drug regimen in RLS (M41L, D67N, K65R, K70R, K103N, V106A/M, Y181C, M184V, G190A, L210W, T215Y/F and K219Q/E) are all present in the shorter RT sequence [8,14-17].

In this study, the potential effect on the prediction of HIVDR by submitting a short RT sequence from amino acid 41 to 238 to the virco^®^TYPE and Stanford resistance interpretation algorithms was investigated through an *in silico *analysis. It was not our intention to compare the performance of virco^®^TYPE versus Stanford.

## Materials and methods

### Amplification of a short RT sequence useful in HIVDR testing

As of today, HIV resistance testing is based on amplifying and sequencing of the viral protease and reverse transcription genes. This requires multiple rounds of amplification and at least 6-8 sequencing reactions. For RLS, we assumed that a cost-reduction could be implemented by sequencing a short RT region. Amplification of this short RT region (codon 41-238) is feasible using a one-step single round amplification followed by a simplified sequencing protocol. Proof of principle for this cost-reduction approach is available [18].

### Virco database analysis

A total of 125,323 full length RT sequences (codon 1-400) were retrieved from the Virco database. For all these sequences, virco^®^TYPE interpretations were generated for the paired full-length RT (codon 1-400) and short RT sequences (codon 41-238) on 8 FDA-approved RT inhibitors commonly used in RLS [6] (lamivudine = 3TC, abacavir = ABC, zidovudine = AZT, stavudine = d4T, didanosine = ddI, tenofovir = TDF, efavirenz = EFV and nevirapine = NVP). A similar approach on non-B subtypes (n = 17,131) was used for the Stanford HIVDR interpretation algorithm.

### virco^®^TYPE HIVDR interpretation tool

*virco^®^TYPE *calculates the phenotypic drug susceptibility from a genotype, based on a linear regression model [19]. The phenotypic drug susceptibility is expressed as a fold change (FC) i.e. the ratio of inhibitory concentration 50% (IC_50_) of a patient-derived sample to the IC_50 _value of a reference strain (IIIB). *virco^®^TYPE *provides a data-driven identification of mutations affecting FC and the magnitude of their effect [19]. The calculated FC per drug is interpreted using cut-off values. The virco^®^TYPE report uses clinical cut-offs (CCOs), where available [20]. Clinical cut-offs are used to facilitate the interpretation of fold change and drug resistance. They represent thresholds on the fold change continuum to indicate loss in clinical drug activity due to resistance. These cut-offs are determined based on observational studies in treated patients. When the calculated FC falls below the lower CCO, a maximal response (MA) to treatment with that drug is predicted, whereas a minimal response (MI) is expected if the FC falls above the higher CCO value. A calculated FC that falls between the lower and higher CCO predicts reduced response (RE). When CCOs are not available for a particular drug (EFV and NVP), biological cut-offs (BCOs) are used. A biological cut-off is based on laboratory observations of viruses derived from treatment naïve patients, and gives an indication of the normal range of in vitro susceptibility of wild-type viruses. The virus is predicted to be susceptible (S) or resistant (R) to a specific drug when the calculated FC is below or above the BCO, respectively [21].

In this analysis virco^®^TYPE VPT4.3.00 was used, with the clinical and biological cut-offs currently in use on the virco^®^TYPE report [20]. The optimal sequence length for virco^®^TYPE analysis is from codon 1 to 99 of the protease region and from codon 1 to 400 of the RT region. The minimal accepted sequence lengths are from codon 10 to 95 and from codon 41 to 238 for protease and RT respectively. Any missing sequence length should be filled with "***" or a reference strain sequence. The virco^®^TYPE linear regression model then calculated the resistance profile.

In this study the output from full RT sequences (codon 1-400) were compared to the resistance prediction of short RT sequences (codon 41-238), whereby the protease gene and RT codon 1-40 were replaced by the HXB2 reference strain sequence.

### Stanford HIVDR interpretation tool

The Stanford HIV database interpretation algorithm is a qualitative HIVDR interpretation tool that assigns a mutation penalty score to each HIV mutation that is, according to published studies, associated with drug resistance[22]. The total score for a drug is derived by adding up the scores of each mutation associated with HIVDR to that drug. The interpretation tool subsequently reports one of the following levels of inferred drug resistance: susceptible (S), potential low-level resistant (pLR), low-level resistant (LR), intermediate resistant (I) and high-level resistant (R) [22]. To simplify the analysis, pLR was regarded as susceptible and LR was interpreted as intermediate. Stanford algorithm version 5.0.0 was used in this analysis.

In contract to virco^®^TYPE, Stanford has no restrictions on the sequence length input. For the Stanford analysis the output from full RT sequences (codon 1-400) were compared to the resistance prediction of short RT sequences (codon 41-238).

### Pair wise comparisons between HIVDR calls generated from full RT and short RT

**Figure 1:**
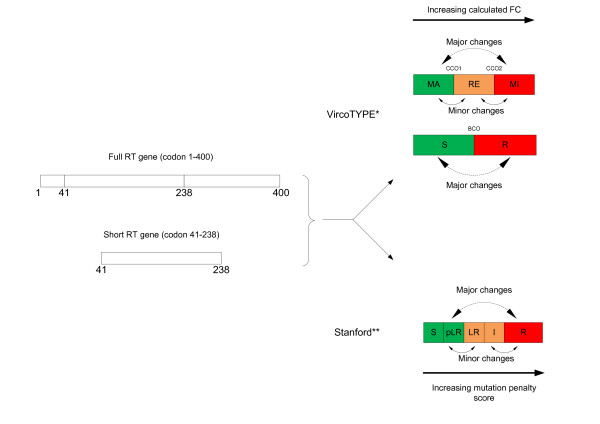
**Representation of the definition of minor and major changes in predicted HIVDR calls between full RT and short RT sequences **. * MA: maximal response; RE: reduced response; MI: minimal response; CCO1: lower clinical cut-off; CCO2: upper clinical cut-off; S: susceptible; R: resistant; BCO: biological cut-off ** S: susceptible; pLR: potential low-level resistant; LR: low-level resistant; I: intermediate resistant; R: high-level resistant

A pair wise comparison of the predicted HIVDR profile (or resistance call) for each full-length and short RT sequence pair was performed for both the virco^®^TYPE and Stanford HIVDR interpretation algorithms. Changes in resistance calls between the full-length RT and the short RT sequence were categorized in major and minor call changes. Major HIVDR call changes are defined as a switch from S to R and MI to MA, or vice versa. Minor call changes include a switch from RE to MA, RE to MI, I to R and I to S, or vice versa (Figure 1). A non-inferiority approach with a concordance limit of 95% and two-sided 95% confidence intervals was used to show if at least 95% of the HIVDR calls based on the short RT sequence (codon 41-238) were concordant with HIVDR calls based on the standard RT sequence (codon 1-400).

## Results

**Figure 2:**
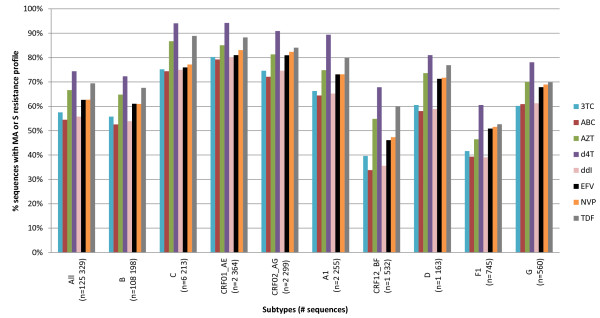
**Dataset, based on subtype and drug-specific full-length RT HIVDR profile by virco^®^TYPE**. The subtypes are arranged by decreasing prevalence of in the Virco database. MA: maximal response, S: susceptible

The dataset used for this analysis contained 125,329 RT sequences. Only HIV subtypes with at least 500 sequences in the database were included for analysis. The majority of sequences were derived from subtype B viruses (n = 108,198), but other non-B subtypes were also represented (n = 17,131). An assortment of 'sensitive' (S or MA) and 'resistant' (RE, MI and R) profiles towards different drugs was observed. The majority of the subtype B sequences were susceptible to RT inhibitors ranging from 52.6% (ABC) to 72.3% (d4T). Due to the delayed introduction of ART in RLS, the proportion of 'sensitive' profiles among the non-B subtypes is higher with the exception of the rare subtypes F1 and CRF12_BF. For the latter two subtypes, specific collaborations had been set up to obtain resistant viruses to enrich the Virco database. A descriptive dataset distribution is depicted in Figure 2.

The HIVDR call changes between full RT and short RT were analyzed per drug in two groups: group 1: sequences that were attributed a 'susceptible' profile (MA or S), based on virco^®^TYPE analysis of the full RT sequence; and group 2: sequences that were attributed a 'resistant' profile (RE, MI or R), based on virco^®^TYPE analysis of the full RT sequence.

### Sequences interpreted by virco^®^TYPE

The *virco^®^TYPE *interpretation based on a full length RT sequence (codon 1-400) was compared to the prediction based on the shortened RT sequence (codon 41-238).

**Figure 3:**
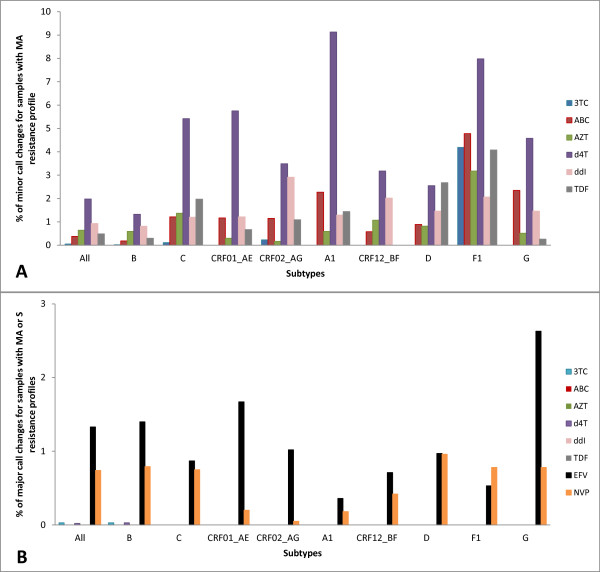
**virco^®^TYPE call changes between full length and short RT HIVDR interpretations for sequences with a 'susceptible' profile, based on full RT interpretation (group 1)**. A. Minor call changes. Minor changes are not possible for drugs with a BCO only as a shift can only occur from susceptible to resistant (or vice versa), which is a major call change; therefore EFV and NVP are not depicted in this graph. B. Major call changes MA: maximal response, S: susceptible

Figure 3a shows that in the 'susceptible' group (group 1) the minor call changes remained below 2%, when all subtypes were pooled together. Subtype-specific analysis demonstrated that at least 95% of HIVDR call-concordance was observed for the majority of the drugs with the exception of d4T. However, across the different drugs, subtype F1 showed a higher proportion of minor call changes, ranging from 3.2% for AZT to 8.0% for d4T. Across subtypes, most minor changes were observed for d4T, ranging from 1.3% for subtype B to 9.1% for subtype A1.

Less than 1.3% major call changes were detected when all sequences from the 'susceptible' group were analyzed. However, 2.6% of the subtype G sequences showed major changes for EFV (Figure 3b).

The analyses for subtype F1 (3TC, ABC and TDF) and subtype G (d4T) were inconclusive. This can be explained by the smaller sample size for subtype F1 (N = 745) and G (N = 560) as compared to the other subtypes (N >1000).

In the other analyses, comparisons between the HIVDR calls based on short and full length RT sequences were concordant in at least 95% of the cases, except for d4T in subtype A1, C, CRF01_AE and F1, with concordance values of 90.87% (95% CI 90.25-91.49%), 94.58% (95% CI 94.30-94.86%), 94.25% (95% CI 93.78-94.72%), 92.02% (95% CI 90.80-93.25%) respectively. Of note, all discordances were caused by minor call changes.

**Figure 4:**
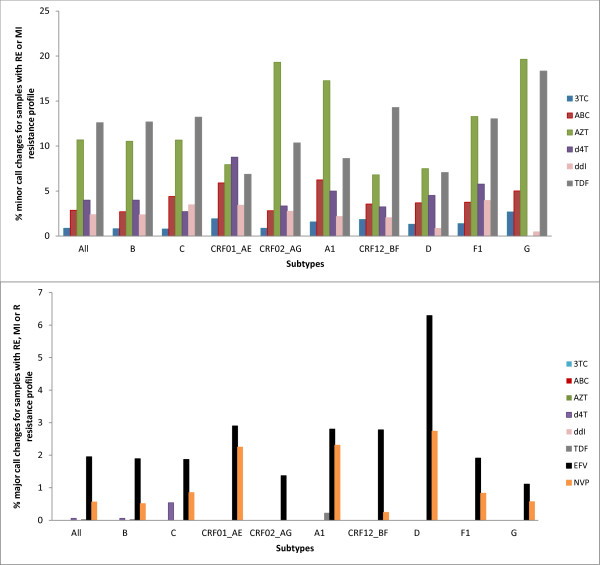
**virco^®^TYPE call changes between full length and short RT HIVDR interpretations for sequences with a 'resistant' profile based on full RT interpretation (group 2)**. A. Minor call changes. Minor changes are not possible for drugs with a BCO only as a shift can only occur from susceptible to resistant (or vice versa), which is a major call change; therefore EFV and NVP are not depicted in this graph. B. Major call changes RE: reduced response, MI: minimal response, R: resistant

As expected, the proportion of call changes increased in the group of 'resistant' samples (group 2), see Figure 4. Overall, there were fewer than 12.6% minor call changes but subtype-specific call changes of up to 19.6% were detected for AZT on subtype G sequences (Figure 4a).

The highest number of major call changes in group 2 were seen among the subtype D samples for NVP (2.7%) and EFV (6.3%), see Figure 4b for more details.

Due to small sample sizes the analyses were inconclusive for the following groups: d4T (subtype A1, D and F1), subtype G (ABC) and CRF01_AE (ABC). Non-inferiority analysis in the remaining groups revealed an inferior HIVDR prediction when using short RT sequences for ABC in subtype A1 sequences, d4T in CRF01_AE, EFV in subtype D, AZT and TDF for all subtypes. As previously observed, all discordances were caused by minor call changes, with the exception of TDF on subtype A1 and B sequences, whereby just a small subset of call changes was of the major type (0.22% and 0.02%, respectively).

### Sequences interpreted by Stanford

The Stanford HIVDR interpretation algorithm was applied only to the non-B sequences (n = 17,131). Neither minor nor major call changes were observed for 3TC, ABC, d4T ddI and TDF. The HIVDR calls for the remaining drugs (AZT, EFV and NVP) changed only in a few cases, with all changes being minor. For AZT, 7 sequences (0.04%) gave a different result when the short RT sequence was submitted to Stanford. The HIVDR level only changed in two sequences (0.01%) for EFV and in 13 sequences (0.08%) for NVP.

## Discussion

There is an increased need for affordable and robust HIV monitoring tools in RLS, including point-of-care VL assays and simplified HIVDR testing protocols. Attempts are being made to simplify currently available technologies in order to make them more accessible for RLS. This study evaluated the use of reducing the sequence length used to interpret HIVDR patterns.

The use of the shorter RT sequences in the virco^®^TYPE HIVDR interpretation tool was not inferior to the full RT sequence for most drugs. An inferior HIVDR interpretation in more than 5% of the cases was detected only for d4T (subtype A1, C, F1 and CRF01_AE) in the group of 'sensitive' sequences. These HIVDR interpretation changes were caused by minor changes: from fully susceptible as predicted by the full RT sequence to reduced susceptibility as predicted by the short RT sequence. Moreover, recent WHO treatment guidelines recommend to phase out the use of d4T as preferred component of first-line treatment [4]. Therefore the clinical impact of HIVDR interpretation for d4T will be limited. In the 'resistant' group, the HIVDR prediction for AZT and TDF is of concern, as more than 5% of the sequences yielded a different HIVDR call for all subtypes when the short RT sequence was submitted to virco^®^TYPE. However, all call changes were minor (from 'reduced response' to 'minimal response', or vice versa), except for TDF for subtype A1 and B samples (0.22% and 0.02% major call changes, respectively). It is therefore unlikely that these HIVDR interpretation changes will have a major clinical impact. Because there is no clinical cut-off available for the NNRTIs NVP and EFV, only major call changes could be observed. The resistance call changes for those two drugs were in most cases limited to less than 3%, which is under our 5% cut-off. Moreover, the clinical relevance for the resistance prediction of these drugs is limited because they are not recommended in second line regimens.

When the sequences were submitted to Stanford, call changes were observed in less than 0.08% of the cases for AZT, EFV and NVP, showing no inferiority of using the short sequence in any of the non-B subtypes (B sequences were not analyzed). Observed HIVDR interpretation differences between full RT and shortened RT sequences in virco^®^TYPE can be explained by the fact that the Virco algorithm includes resistance weight factors for a substantial number of codon positions outside the RT codon 41-238 region which are depicted in Table 1. The Stanford algorithm is based on mutations that all lie within the region of RT codon 41 to 238, except for 333D, 333E and 318F, (see Table 1). The latter three mutations influence HIVDR only towards AZT, EFV and NVP and were only present in 2% of the non-B subtypes (405/17,131). One could argue that part of the observed differences between Stanford and virco^®^TYPE could be explained by the fact that the reference strains, used in Stanford and virco^®^TYPE, are different (consensus B versus HXB2 respectively). However, both reference strains only differ from each other at four positions (codon 122, 214, 376 and 400). Moreover, in most cases, resistance mutations at those four positions would be picked up by both algorithms as they are different from the reference amino acids found in either HXB2 or the consensus B sequence.

**Table 1:** Amino acid positions outside RT codon 41-238 contributing to the HIVDR interpretation algorithms. ROI: region of interest

ROI	ARV drugs	Stanford	virco^®^TYPE
RT codon 1-40	3TC	none	7, 8, 13, 35, 36, 40 (n = 6)
	ABC	none	3, 13, 21, 33, 35, 39, 40 (n = 7)
	AZT	none	none
	d4T	none	3, 13, 33, 35, 36, 40 (n = 6)
	ddI	none	3, 4, 33, 35, 36, 39, 40 (n = 7)
	TDF	none	4, 7, 13, 21, 33, 40 (n = 6)
	EFV	none	16, 20, 22, 27, 28, 31, 33, 34 (n = 8)
	NVP	none	21, 31, 35 (n = 3)

RT codon 239-400	3TC	none	240, 248, 277, 313 (n = 4)
	ABC	none	334, 348 (n = 2)
	AZT	333 (n = 1)	240, 242, 244, 245, 282, 296, 297, 313, 334, 335, 350, 357, 359, 360, 375, 377, 386, 395 (n = 18)
	d4T	none	334, 348, 357, 359 (n = 4)
	ddI	none	348, 359, 360, 395 (n = 4)
	TDF	none	242, 245, 249, 277, 297, 329, 334, 335, 353, 357, 359, 395 (n = 12)
	EFV	318 (n = 1)	240, 241, 243, 244, 245, 250, 251, 257, 271, 272, 274, 282, 283, 286, 292, 297, 313, 317, 318, 329, 333, 334, 335, 338, 339, 348, 353, 356, 357, 358, 365, 366, 369, 370, 371, 375, 376, 377, 379, 381, 382, 385, 386, 390, 393, 394, 395, 400 (n = 48)
	NVP	318 (n = 1)	244, 245, 248, 250, 272, 283, 286, 293, 297, 313, 317, 318, 329, 333, 334, 335, 338, 339, 348, 353, 356, 357, 358, 365, 366, 369, 370, 371, 374, 375, 376, 377, 379, 382, 385, 386, 390, 393, 394, 395, 399, 400 (n = 42)

Overall this study shows that the use of a shorter RT sequence genotype results in >95% concordance with results obtained from full length RT sequences obtained from two routinely used interpretation systems, virco^®^TYPE and Stanford. The results provide initial validation that the simpler shorter genotype can be considered for use in a new ARV-treatment monitoring system for use in RLS.

Nevertheless, this study also has some limitations. Firstly, despite a good representation of non-B subtypes (n= 17,131) in the dataset used, the majority of sequences in this database are subtype B, which is less relevant for RLS. To accommodate this limitation, further analysis on the effect of sequencing a short RT fragment for HIVDR testing of RLS samples accessing first-line regimens will be done in collaboration between ART-A and the PASER (PharmAccess African Studies to Evaluate Resistance) network [23]. Secondly, the treatment data of the patients from which these sequences were derived is missing and therefore we could not make a clear differentiation between the resistance interpretations in a treatment naïve group versus a treatment exposed group. This issue will also be addressed in the future study (mentioned above) as treatment naïve and treatment failing patients will be included. Thirdly, this simplified resistance assay only focuses on assessing resistance in the RT gene, which is relevant for RLS at the moment as most of the patients receive a combination ARV regimen of RT inhibitors only. However, when protease inhibitors will become more readily available in RLS there will be a need to include the protease gene as well.

Although this simplified HIVDR interpretation algorithm still requires a lab infrastructure, skilled personnel and investment in major equipment, it also has several advantages. Firstly, amplification of the short RT region is feasible using a one-step single round amplification protocol [18], which reduces the risk for contamination, minimizes hands-on work and cuts down the reagent cost as only one amplification primer set is needed. Secondly, the sequencing is also simplified by reducing the number of primers from 8 (in Virco's in-house assay) to only 2. Thirdly the analysis time of the obtained short RT sequence is also reduced compared to the analysis of a full RT sequence. The obtained short RT sequence can subsequently be submitted to either Stanford or virco^®^TYPE. However, the biological starting material for this simplified HIVDR algorithm is plasma, which might pose a problem in RLS, as cold-chain transport and deep frozen storage is still a challenge in many places. Therefore, the ART-A team is currently investigating the feasibility of using dried blood spots as a source material to overcome this issue.

In conclusion, this comparative analysis has shown that HIVDR interpretation, based on shorter RT sequence, is not inferior compared to the use of full RT sequences for most of the commonly used HIV RT inhibitors in RLS.

## Competing interests

KS, EVK, BW, KvdB, and LJS are employees of Tibotec-Virco Virology BVBA. The company commercializes HIV drug resistance testing technology on the codon 1-400 RT domain. While the present study does not represent a commercial activity, products using the complete RT codon are commercialized by the company in the western world. However, no commercial activities are planned for RLS specifically. Other authors declare that they have no competing interests.

## Authors' contributions

KS designed the study, performed the analysis and prepared the manuscript. MB assisted in drafting the manuscript. EvC and KvdB performed the analysis. BW took care of the statistical analysis. WS, CW and TRdW and LS assisted in designing the study and provided substantial intellectual content to the manuscript. All authors critically reviewed and approved the final manuscript.
